# Assessment of corticospinal tract remodeling based on diffusion tensor imaging in the treatment of motor dysfunction after ischemic stroke by acupuncture: A meta-analysis

**DOI:** 10.1097/MD.0000000000034618

**Published:** 2023-08-11

**Authors:** Weiming Zhu, Shizhe Deng, Hailun Jiang, Jieying Zhang, Boxuan Li, Qingqing Jia, Zhihong Meng

**Affiliations:** a First Teaching Hospital of Tianjin University of Traditional Chinese Medicine, Tianjin, China; b National Clinical Research Center for Chinese Medicine Acupuncture and Moxibustion, Tianjin, China; c The Second Affiliated Hospital of Shandong University of Traditional Chinese Medicine, Jinan, China.

**Keywords:** acupuncture, corticospinal tract, ischemic stroke, meta-analysis, motor function

## Abstract

To investigate the efficacy of acupuncture in improving motor dysfunction after ischemic stroke (IS) and to investigate the effect of acupuncture on corticospinal tract (CST) remodeling using diffusion tensor imaging.

**Methods:**

Published literature on the effect of acupuncture on CST remodeling after IS using diffusion tensor imaging in the form of randomized controlled trials (RCTs) were systematically retrieved and screened from Cochrane Library, Web of Science, PubMed, Embase, CNKI, CBM, VIP, and Wanfang databases from inception to December 2022. The methodological quality of the included studies was critically and independently evaluated by 2 reviewers using the Cochrane Risk of Bias Assessment Tool for RCTs. The correlated data were extracted using the pre-designed form, and all analyses were performed using Reviewer Manager version 5.4.

**Results:**

Eleven eligible RCTs involving 459 patients were eventually included. The combined evidence results showed that the acupuncture group significantly improved patients’ National Institute of Health stroke scale, Fugl-Meyer Assessment Scale, and Barthel index compared with conventional medical treatment. The acupuncture group significantly promoted remodeling of the CST, as reflected by an increase in fractional anisotropy (FA) throughout the CST [MD = 0.04, 95% CI (0.02, 0.07), *P* = .001], and in addition, subgroup analysis showed that the acupuncture group significantly improved FA in the infarct area compared with conventional medical treatment at around 4 weeks [MD = 0.04, 95% CI (0.02, 0.06), *P* = .0002] and FA of the affected cerebral peduncle [MD = 0.03, 95% CI (0.00, 0.07), *P* = .02]. Also, compared with conventional medical treatment, the acupuncture group significantly increased average diffusion coefficient of the affected cerebral peduncle [MD = −0.21, 95% CI (−0.28, −0.13), *P* < .00001].

**Conclusion:**

The results of the meta-analysis suggest that acupuncture therapy can improve the clinical manifestations of motor dysfunction in patients after IS and advance a possibly beneficial effect on CST remodeling. However, due to the number and quality of eligible studies, these findings need to be further validated in more standardized, rigorous, high-quality clinical trials.

## 1. Introduction

In recent years, stroke has become one of the leading causes of death among Chinese residents, and the lifetime risk of stroke in China is 39.3%, which is 14.4% higher than the global lifetime risk of stroke as estimated by the Global Burden of Disease Working Group. Cerebrovascular disease mortality accounts for 22% of mortality among Chinese residents, and ischemic stroke (IS) accounts for 82% of stroke patients.^[1]^ IS is characterized by acute onset, mainly due to impaired blood circulation, leading to localized ischemic necrosis or softening of brain tissue due to ischemia and hypoxia. Once the onset is often accompanied by irreversible damage to brain tissue, about 75% of patients are left with varying degrees of motor dysfunction,^[2]^ which seriously affects patients’ quality of life and adds a heavy burden to society and patients’ families.^[3]^ In China, patients with motor dysfunction after IS are mostly treated with a rehabilitation program combining Chinese and Western medicine. In recent years, acupuncture has played an important role in the rehabilitation of patients with motor dysfunction after IS, and the clinical use of acupuncture as a complementary or alternative therapy has increased worldwide.^[4–6]^ In 2002, the World Health Organization also recommended acupuncture as a treatment for stroke, and they concluded that acupuncture has some advantages in the treatment of post-stroke patients with dysfunction, such as motor dysfunction, sensory dysfunction, and aphasia. To date, many studies have reported the role and potential mechanisms of acupuncture in improving neurological prognosis in IS. Numerous animal studies have also shown that acupuncture exerts neuroprotective effects in IS, such as promoting neuroregeneration, angiogenesis, and neuroplasticity.^[7]^ Currently, a large number of systematic evaluations have assessed the efficacy of acupuncture for stroke. However, the brain microstructure mechanism of acupuncture intervention in patients with dyskinesia after stroke is still unclear, and the available systematic evaluations have not yet evaluated the effect of acupuncture intervention on corticospinal tract (CST) remodeling in patients with movement disorders. The CST is an important component of the pyramidal system that translates the brain’s conscious intent into observable actions and is particularly important for the motor function of the distal limb.^[8]^ lesions in patients with IS often involve the CST, resulting in compression, injury, or even fracture of the CST, leading to corresponding motor function impairment.^[9,10]^ Therefore, an adequate grasp of the infarct size and CST damage in the clinic can help to make an effective judgment of the degree of motor function impairment in patients.^[11]^ MRI is an important basis for confirming the diagnosis and localization of IS in clinical practice. Among them, Diffusion Tensor Imaging (DTI) can show the morphological changes of the corticospinal tracts by processing visualization, and can accurately quantify the damage of the white matter fiber tracts by fractional anisotropy (FA), average diffusion coefficient (ADC), etc., which can objectively reflect the degree of CST nerve damage in IS patients.^[12,13]^ It has been demonstrated that the results of white matter integrity detection by DTI are reproducible and reliable.^[14]^ Assessment of CST integrity by DTI has also been shown to be an objective assessment and reliable in individuals with IS.^[15,16]^ The DTI has been widely used to analyze the correlation between white matter changes and functional responses in stroke,^[17]^ to measure corticospinal integrity after stroke,^[18]^ to assess white matter damage in patients who have neglected stroke,^[19]^ and to predict the outcome of motor function after stroke rehabilitation.^[20]^

Therefore, this paper explores a clinical study of acupuncture with the aid of the DTI technique for the treatment of patients with motor dysfunction in IS. In contrast to previous systematic evaluations, the present study focused on assessing corticospinal tract remodeling after acupuncture treatment of IS. With this, the clinical efficacy of acupuncture in treating patients with motor dysfunction in IS relative to single rehabilitation treatment was evaluated, which can further provide a reference for the clinical application of acupuncture in treating IS. We present the following article by the PRISMA reporting checklist (see Table S1, Supplemental Digital Content, http://links.lww.com/MD/J425, which illustrates the checklist item of the PRISMA 2020 Checklist).

## 2. Materials and methods

To increase the transparency and quality of systematic evaluation reports, this research complied with PRISMA 2020 statement and has been registered in PROSPERO (registration number: CRD42021283638).

### 2.1. Inclusion and exclusion criteria

#### 2.1.1. Types of research.

Randomized controlled trials based on diffusion tensor imaging to assess the efficacy of acupuncture in the treatment of patients with motor dysfunction after IS were strictly included, regardless of the language of publication. Specific and accurate data are available for analysis.

#### 2.1.2. Object of study.

All subjects met the diagnostic criteria for IS established by the Chinese Medical Association,^[21]^ without differentiating between age, sex, race, and region.

#### 2.1.3. Intervention Measures.

The control group was treated with conventional medical therapy. Conventional medical treatment included symptomatic, supportive, and preventive treatment of complications such as thrombolysis or defibrillation, anticoagulation, antiplatelet aggregation, cerebral metabolic protection, reduction of cerebral edema, improvement of cerebral circulation, and reduction of intracranial pressure, also with herbal medicine, moxibustion, or rehabilitation training. The experimental group was given acupuncture treatment based on the control group, including manual acupuncture, electroacupuncture, scalp acupuncture, and tongue acupuncture.

#### 2.1.4. Exclusion criteria.

Studies that were not rigorous, had incomplete data, or had significant errors and could not be statistically analyzed were excluded. Studies with experimental or control groups for other interventions, animal experiments, empirical reports, conference papers, and duplicate publications that were not available in the full text were excluded.

#### 2.1.5. Observation indexes.

The primary regression indicators are as follows: FA. Secondary regression indicators are as follows: ADC; National Institute of Health stroke scale (NIHSS); Fugl-Meyer Assessment Scale (FMA); and Barthel index (BI).

### 2.2. Retrieval strategy

Computer searches randomized controlled trials of DTI-based acupuncture for the treatment of motor dysfunction after IS from Cochrane Library, Web of Science, PubMed, Embase, CBM, CNKI, VIP, and Wanfang databases from database creation to December 2022. To ensure the comprehensiveness and completeness of the literature, a combination of subject terms and free terms was used for the literature search. The search terms used in combination and the search strategy are listed in Table S2, Supplemental Digital Content (http://links.lww.com/MD/J426,which illustrates the search strategy and search terms).

### 2.3. Literature screening and data extraction

According to the flowchart of PRISMA, 2 researchers independently screened the literature based on inclusion and exclusion criteria and independently extracted relevant data (author name, year of publication, sample size, age, duration of treatment, acupuncture duration, frequency of acupuncture, acupuncture points, and outcome indicators) and cross-checked them after extraction. In case of disagreement, the 3rd researcher will assist in the resolution.

### 2.4. Methodological quality evaluation

Two investigators evaluated the quality of the included literature according to the requirements of the risk of bias assessment tool entries in the Cochrane System Reviewer Manual 5.1.0, including the following 7 entries: generation of randomized sequences; whether the allocation was concealed in rows; whether blinding was used for patients and staff; whether the study outcomes were evaluated blindly; whether the outcome data were complete; and whether there was selective reporting; and whether there was another bias. Judgments were made on the literature based on evaluation criteria for 3 types of quality outcomes: high, low, and uncertain.^[22]^ All of the above were independently evaluated by 2 investigators on the included literature, and if there was disagreement, the disagreement was resolved by discussion and decision by Prof Zhihong Meng.

### 2.5. Statistical treatments

RevMan 5.4 software (developed by the UK’s International Cochrane Collaboration) was performed for data analysis.^[23]^ We calculated the estimates for the clinical outcomes and their 95% confidence intervals (CIs). when the measurement method and measurement unit are consistent in continuous variables, mean difference (MD) was adopted as the effect analysis statistics; if not, the standard mean difference was used. Relative risks were used as a practical measure for dichotomous variables. The Chi-square test was used to assess statistical heterogeneity (*P* < .1 is considered as significant statistical heterogeneity) between data of included trials and *I*^2^ test was used to determine the degree of heterogeneity (*I*^2^ ≥ 50% is considered as high heterogeneity; < 50% was considered unapparent). The random-effect model was selected when heterogeneity is high, and the fixed-effect model was applied when heterogeneity is acceptable. Sensitivity analysis or subgroup analysis was used to explore sources of heterogeneity. The Funnel plot was created to evaluate the publication bias of each included literature.

## 3. Result

### 3.1. Literature search

**Figure 1.**
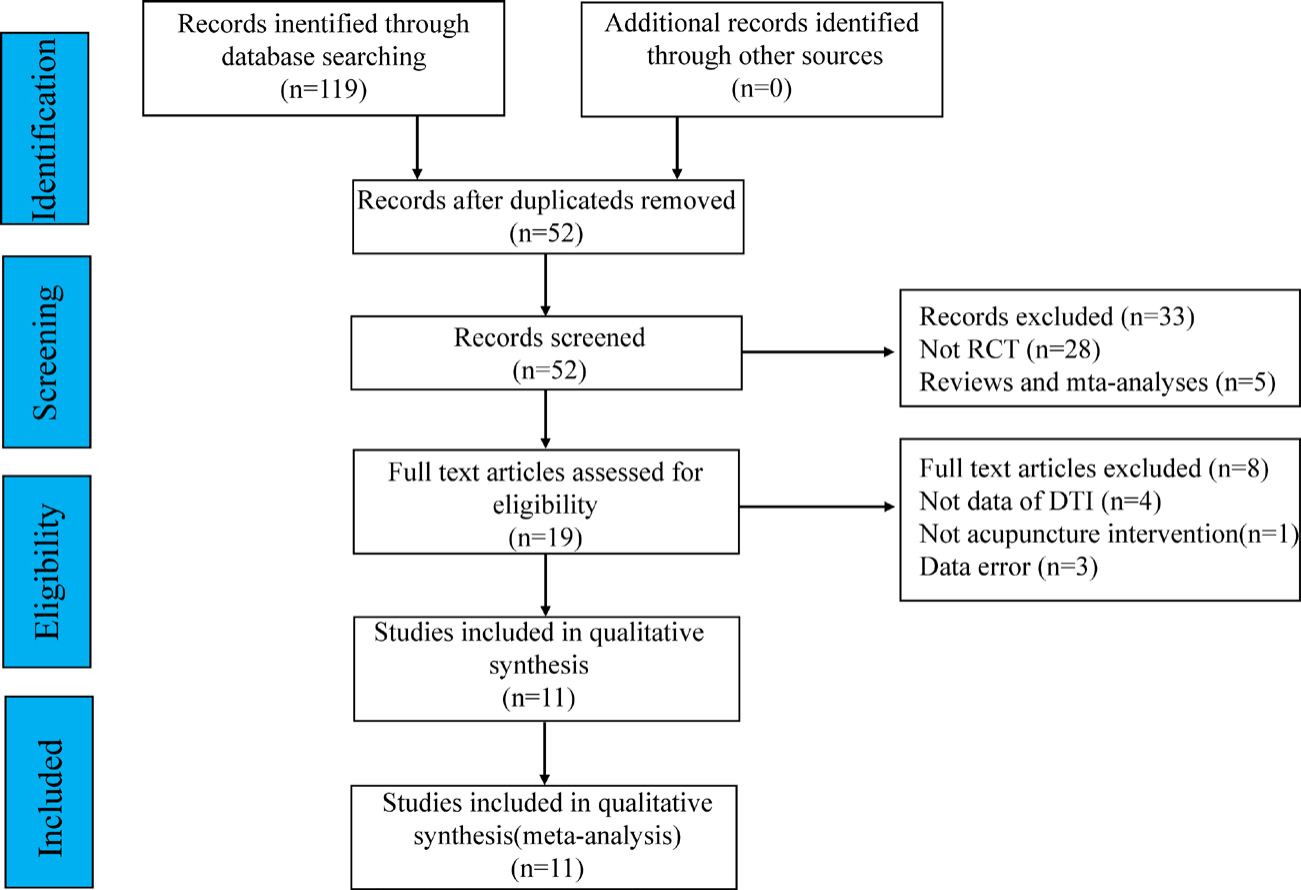
Flowchart of literature retrieval and screening. DTI = diffusion tensor imaging, RCT = randomized controlled trial.

A preliminary search of 119 studies was conducted, including 19 English and 100 Chinese studies. After removing duplicate studies and reading the titles, abstracts, and full text, the remaining 108 studies were excluded. Eleven RCTs were finally included. The flow chart of the studies screening and the results are shown in Figure 1.

### 3.2. Basic characteristics of studies in the literature

11 eligible studies involving 459 patients, 10 of which were published in Chinese databases and 1 in English databases.^[24–34]^ There were 232 cases in the treatment group and 227 cases in the control group. All studies had clear inclusion and exclusion criteria and reported comparable baselines for the treatment and control groups. The intervention in all studies was acupuncture combined with conventional medical treatment in the treatment group, and the intervention in the control group was conventional medical treatment. For the observed outcome indicators, 4 studies performed NIHSS, 5 studies performed BI, 3 studies performed FMA, 8 studies observed FA, 5 studies observed ADC, and no adverse effects were observed in all studies. The basic characteristics of the 11 eligible papers are shown in Table 1.

**Table 1:** Basic characteristics of literature research.

Study ID	Sex (M/F)		Sample size		Age (yr)		Duration	Follow-up time	Random method	Blinding	Intervention	Control	Outcomes
	AG	CG	AG	CG	AG	CG							
Yuan C, 2013	9/6	10/4	15	14	49.7 ± 9.4	56.9 ± 6.7	2 wk	3 mo	Random	NR	Acupuncture + control	Medical treatment + Rehabilitation training	③④
Liao SQ, 2015	39/21	40/20	60	60	60.37 ± 17.13	61.82 ± 10.28	1 mo	NR	Table of random digit	Triple blind	Acupuncture + control	Medical treatment	①②
Jiang SZ, 2017	8/2	8/3	10	11	68.4 ± 4.5	57.1 ± 9.8	2 wk	NR	Table of random digit	Double blind	Acupuncture + control	Medical treatment	③⑤
Liu Y, 2020	16/13	14/15	29	29	61.35 ± 5.14	60.27 ± 6.37	2wk	NR	Table of random digit	NR	Acupuncture + control	Medical treatment	①②⑤
Yang FX, 2021*	15/8	14/9	23	23	63.60 ± 13.42	59.73 ± 11.5	4 wk	NR	Table of random digit	NR	Acupuncture + control	Medical treatment	①②④
Yang FX, 2021	14/13	15/12	27	27	63.36 ± 19.07	61.24 ± 16.51	4 wk	NR	Table of random digit	NR	Acupuncture + control	Medical treatment	④⑤
Shen YX, 2013	8/2	8/2	10	10	55.00 ± 13.14	55.80 ± 14.38	6 wk	NR	Table of random digit	NR	Acupuncture + control	Medical treatment	①②③⑤
Li J, 2020	11/7	9/9	18	18	62.00 ± 12.29	63.22 ± 11.16	1 mo	NR	Random	NR	Acupuncture + control	Medical treatment	①
Liu L, 2015	9/5	8/3	14	11	62.06 ± 7.07	65.43 ± 4.65	3 mo	NR	Random	Double blind	Acupuncture + control	Rehabilitation training	①
Wu ZJ, 2015	9/7	8/6	16	14	63.06 ± 7.07	65.44 ± 4.65	4 wk	NR	Table of random digit	Single blind	Acupuncture + control	Rehabilitation training	①
Shen YX, 2012	NR		10	10	55.00 ± 13.14	55.80 ± 14.38	2 wk	8 wk	Random	NR	Acupuncture + control	Medical treatment	①②③⑤

Control: Conventional medical treatment included symptomatic, supportive, and preventive treatment of complications such as thrombolysis or defibrillation, anticoagulation, antiplatelet aggregation, cerebral metabolic protection, reduction of cerebral edema, improvement of cerebral circulation, and reduction of intracranial pressure, also with herbal medicine, moxibustion, or rehabilitation training.

ADC = apparent diffusion coefficient, AG = acupuncture group, BI = Barthel index, CG = control group, F = female, FA = fractional anisotropy, FMA = Fugl-Meyer Assessment scale, M = male, NIHSS = National Institute of Health stroke scale, NR = not report.

### 3.3. Risk of bias assessment

**Figure 2.**
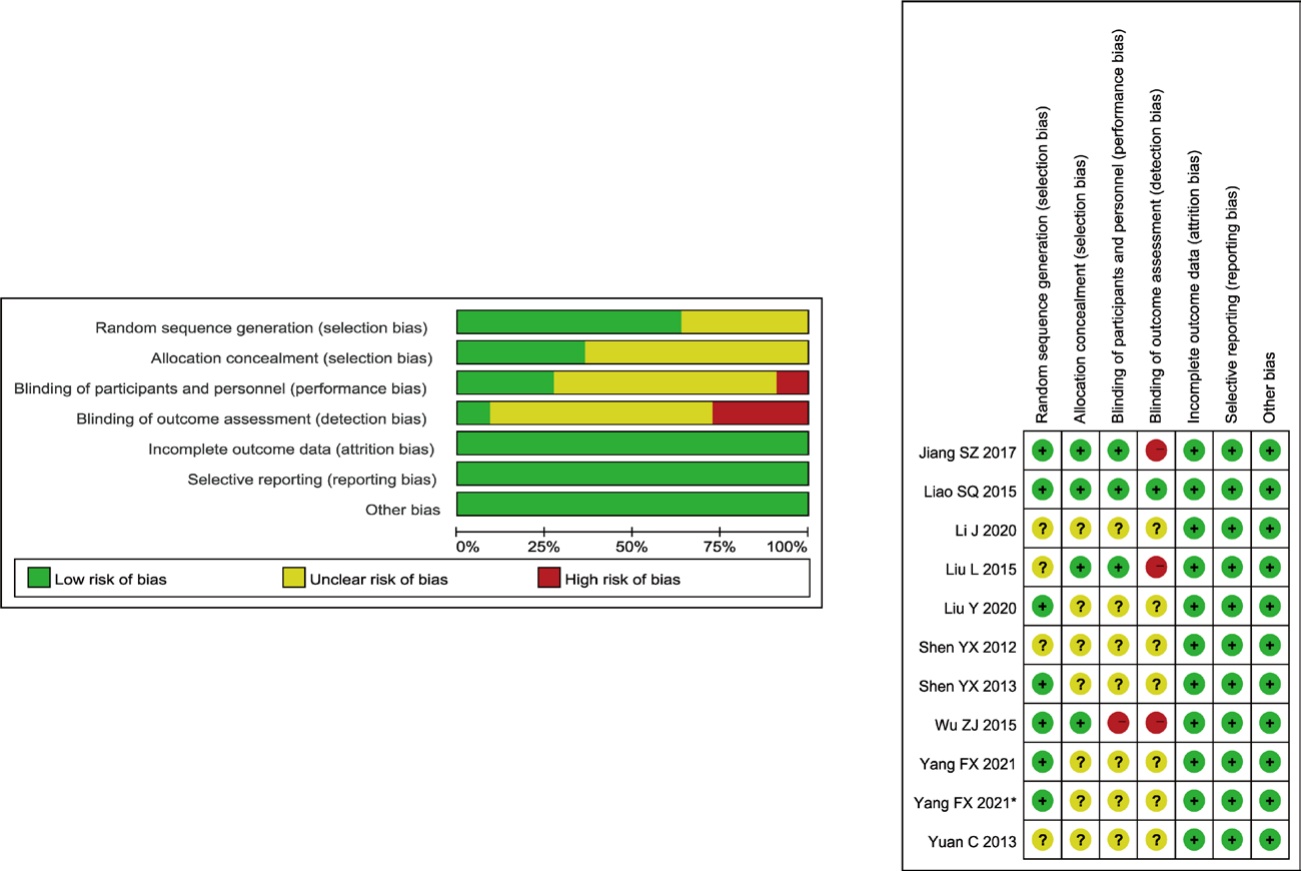
Risk of bias assessment of included randomized controlled trials.

All eligible studies were randomized controlled trials, among which 7 studies^[25–30,33]^ described the methods of the randomization process in detail, taking the random number table as the specific method. The remaining 4 studies^[24,31,32,34]^ were reported as “randomized” but were not specifically described. One study^[25]^ used central randomization to generate random grouping sequences. 3 studies^[26,30,32]^ put block codes into envelopes and sealed them. The remaining 7 studies did not mention allocation hiding. Only one study^[25]^ adopted the triple-blind method, 2 studies^[26,32]^ adopted the double-blind method, and one study^[33]^ adopted the single-blind method. There was no missing outcome data or selective reporting bias in all literature, rated as “low risk.” No obvious other risks were found in all the literature, so the other bias was evaluated as “low risk.” Specific risk assessment information of bias is shown in Figure 2.

### 3.4. Interventions

According to the STRICTA guideline,^[35]^ the needle stimulation included manual acupuncture (8 studies) and electroacupuncture (3 studies). The types of acupuncture therapy included head acupuncture (9 studies), and body acupuncture (2 studies). For acupuncture points, all 11 studies selected acupuncture points according to TCM theory and the meridian system. A total of 39 acupoints were mentioned in 11 studies, 103 times. Among them, DU 20 (Baihui, 8/11) is the most commonly used acupuncture point. Other top 2 used acupoints included SP6 (Sanyinjiao, 7/11), and ST36 (Zusanli, 6/11). 1 scalp acupuncture area was involved in 3 studies for 3 times involving MS6 (anterior oblique line of vertex-temporal, the line joining anterior EX-HN1 and GB6 Xuanli). In 6 studies, acupuncture-induced reactions were described as “de qi.” The detailed interventions of the 12 studies are shown in Table 2.

**Table 2:** Intervention details.

Study ID	AG Intervention	CG Intervention	Duration	Follow-up time	Needle session	Needle type	Needle frequency	Needle response	Acupoint
Yuan C, 2013	MA + control	Medical treatment + Rehabilitation training	2 wk	3 mo	10	Body acupuncture. scalp acupuncture	5 times per week	NR	Shangxing (DU23), Baihui (DU20), Sishencong (EX-HN1), Jianwaishu (SI14), Jianliao (SJ14), Binao (LI14), Waiguan (SJ5), Hegu (LI4), Neiguan (PC6), Quchi (LI11), Huantiao (GB30), Fengshi (GB31), Liangqiu (ST34), Taichong (LR3), Sanyinjiao (SP6), Zusanli (ST36)
Liao SQ, 2015	EA + control	Medical treatment	4 wk	NR	20	Body acupuncture	5 times per week	De qi	Quchi (LI11), Waiguan (SJ5)
Jiang SZ, 2017	MA + control	Medical treatment	2 wk	NR	10	Scalp acupuncture	5times per week	NR	MS6
Liu Y, 2020	MA + control	Medical treatment	2 wk	NR	14	Body acupuncture, scalp acupuncture	7 times per week	De qi	Hegu (LI4), Waiguan (SJ5), Shousanli (LI10), Quchi (LI11), Jianyu (LI15), Jiexi (ST41), Xuehai (SP10), Zusanli (ST36), Yangliquan (GB34), Sanyinjiao (SP6), Huantiao (GB30), Liangqiu (ST34), Shenting (DU24)
Yang FX, 2021*	MA + control	Medical treatment	4 wk	NR	12	Body acupuncture. scalp acupuncture	6 times per week	NR	Baihui (DU20), Shuigou (DU26), Chengjiang (RN24), Guanyuan (RN4), Qihai (RN6), Zhongwan (RN12), Shenting (DU24), Mingmen (DU4), Jianyu (LI15), Chize (LU5), Hegu (LI4), Houxi (SI3), Fengshi (GB31), Weizhong (BL40), Zusanli (ST36), Xuanzhong (GB39), Taichong (LR3)
Yang FX, 2021	MA + control	Medical treatment	4 wk	NR	12	Body acupuncture. scalp acupuncture	6 times per week	NR	Baihui (DU20), Shuigou (DU26), Chengjiang (RN24), Guanyuan (RN4), Qihai (RN6), Zhongwan (RN12), Shenting (DU24), Mingmen (DU4), Jianyu (LI15), Chize (LU5), Hegu (LI4), Houxi (SI3), Fengshi (GB31), Weizhong (BL40), Zusanli (ST36), Xuanzhong (GB39), Taichong (LR3)
Shen YX, 2013	MA + control	Medical treatment	6 wk	NR	14	Body acupuncture. scalp acupuncture	7 times per week	De qi	Shangxing (Du23), Baihui (Du20), Yintang (EX-HN3), Neiguan (PC6), Sanyinjiao (SP6), Jiquan (HT1), Chize (LU5), Weizhong (BL40)
Li J, 2020	MA + control	Medical treatment	4 wk	NR	28	Body acupuncture. scalp acupuncture	7 times per week	De qi	Baihui (DU20), Yintang (EX-HN3), Jianyu (LI15), Quchi (LI11), Waiguan (SJ5), Hegu (LI4), Futu (ST32), Zusanli (ST36), Sanyinjiao (SP6), Taichong (LR3)
Liu L, 2015	EA + control	Rehabilitation training	3 mo	NR	10	Body acupuncture. scalp acupuncture	5 times per week	De qi	Baihui (DU20), MS6, Jianyu (LI15), Quchi (LI11), Waiguan (SJ5), Zusanli (ST36), Yangliquan (GB34), Sanyinjiao (SP6)
Wu ZJ, 2015	EA + control	Rehabilitation training	4 wk	NR	10	Body acupuncture. scalp acupuncture	5 times per week	De qi	Baihui (DU20), MS6, Jianyu (LI15), Quchi (LI11), Shousanli (LI10), Zusanli (ST36), Yangliquan (GB34), Sanyinjiao (SP6)
Shen YX, 2012	MA + control	Medical treatment	2 wk	8 wk	14	Body acupuncture. scalp acupuncture	7 times per week	NR	Shangxing (Du23), Baihui (Du20), Yintang (EX-HN3), Neiguan (PC6), Sanyinjiao (SP6)

Control = Conventional medical treatment included symptomatic, supportive, and preventive treatment of complications such as thrombolysis or defibrillation, anticoagulation, antiplatelet aggregation, cerebral metabolic protection, reduction of cerebral edema, improvement of cerebral circulation, and reduction of intracranial pressure, also with herbal medicine, moxibustion, or rehabilitation training.

AG = acupuncture group, CG = control group, EA = electroacupuncture, MA = manual acupuncture.

### 3.5. Synthesis of outcome

#### 3.5.1. NIHSS.

**Figure 3.**
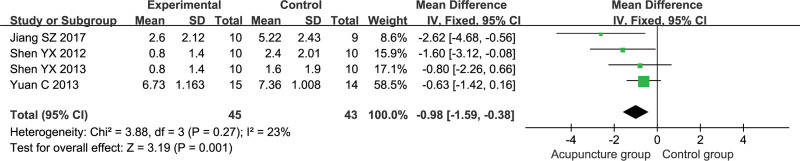
Meta-analysis of the National Institute of Health stroke scale.

Four studies reported NIHSS including 45 patients in the acupuncture group and 43 patients in the control group.^[24,26,30,34]^ Meta-analysis was performed using a fixed-effects model (*P* = .27, *I*^2^ = 23%). The results are shown in Figure 3, where the acupuncture group improved NIHSS better than the control group [MD = −0.98, 95% CI (−1.59, −0.38), *P* = .001].

#### 3.5.2. BI.

**Figure 4.**
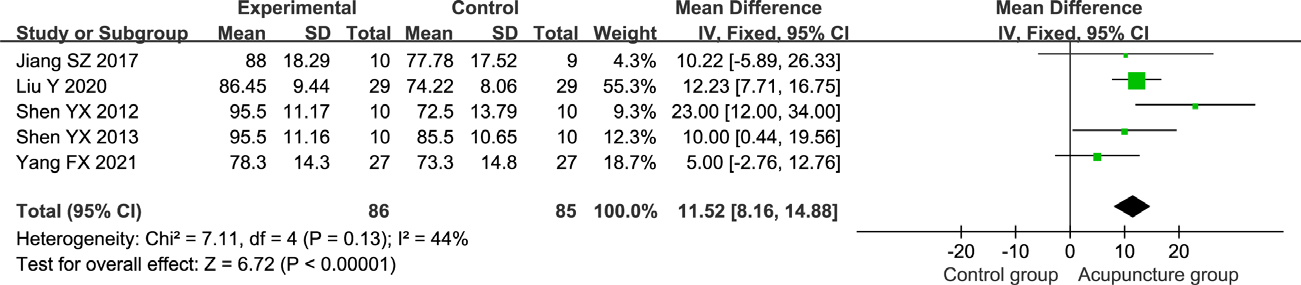
Meta-analysis of the Barthel index.

Five studies reported Barthel Index, including 86 patients in the acupuncture group and 85 patients in the control group.^[26,27,29,30,34]^ Meta-analysis was performed using a fixed-effects model (*P* = .13, *I*^2^ = 44%). The results are shown in Figure 4, where the acupuncture group improved BI better than the control group [MD = 11.52, 95% CI (8.16, 14.88), *P* < .00001].

#### 3.5.3. FMA.

**Figure 5.**
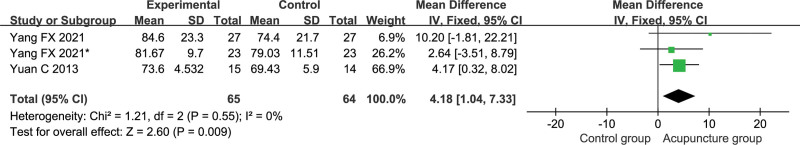
Meta-analysis of the Fugl-Meyer Assessment Scale.

Three studies reported FMA, including 65 patients in the acupuncture group and 64 patients in the control group.^[24,28,29]^ Meta-analysis was performed using a fixed-effects model (*P* = .55, *I*^2^ = 0%). The results are shown in Figure 5, where the acupuncture group improved FMA better than the control group [MD = 4.18, 95% CI (1.04, 7.33), *P* = .009].

#### 3.5.4. FA.

**Figure 6.**
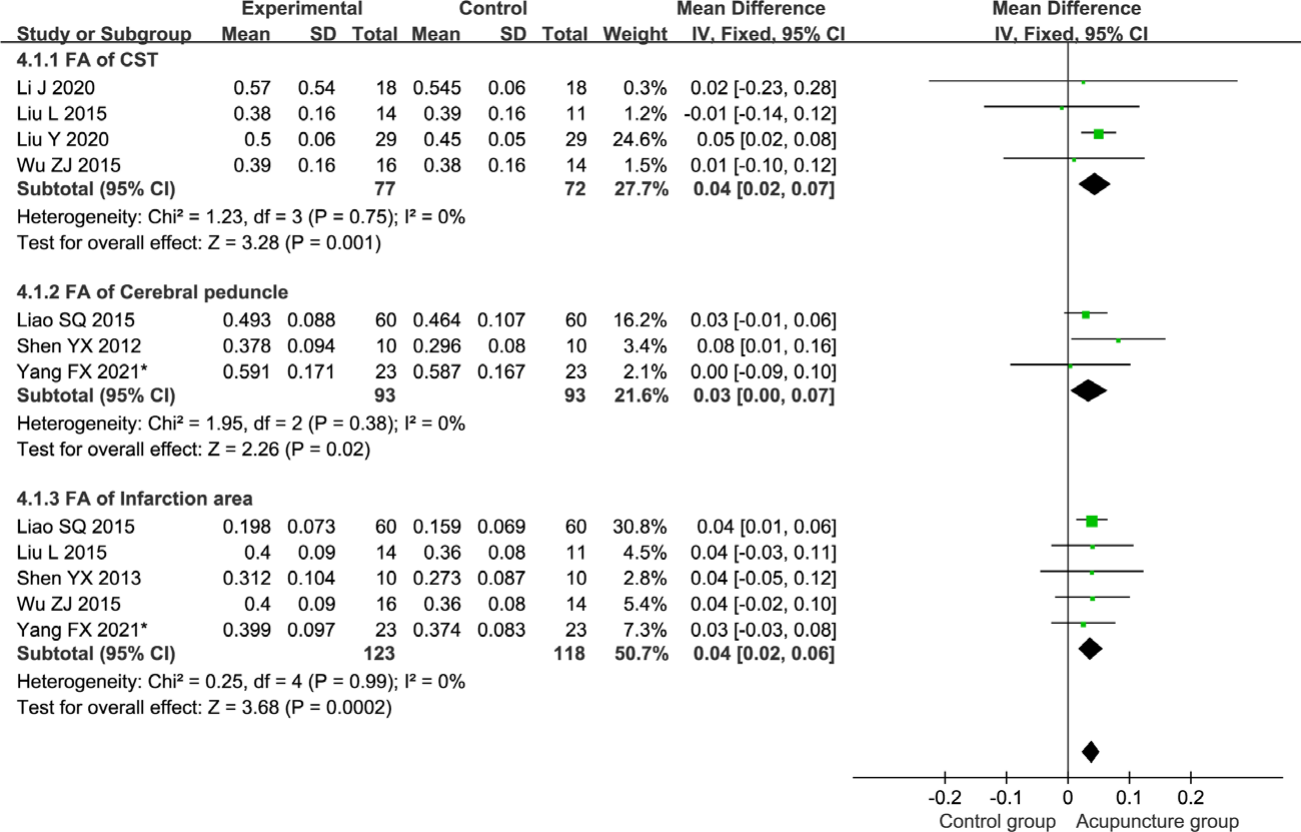
Subgroup analysis of the fractional anisotropy. CST = corticospinal tract, FA = fractional anisotropy.

Four studies reported the mean FA at every 100 equidistant points throughout the bilateral CST, including 77 patients in the acupuncture group and 72 patients in the control group.^[27,31–33]^ Meta-analysis was performed using a fixed-effects model (*P* = .75, *I*^2^ = 0%). The results are shown in Figure 6, where the acupuncture group improved the FA of bilateral CST fiber tracts better than the control group [MD = 0.04, 95% CI (0.02, 0.07), *P* = .001]. Three studies reported FA on the affected cerebral peduncle, including 93 patients in the acupuncture group and 93 patients in the control group.^[25,28,34]^ Meta-analysis was performed using a fixed-effects model (*P* = .38, *I*^2^ = 0%). The results are shown in Figure 6, and the test group improved the FA values of the affected cerebral peduncle better than the control group [MD = 0.03, 95% CI (0.00, 0.07), *P* = .02]. 5 studies reported FA of the CST across the infarct area, including 93 patients in the test group and 93 patients in the control group.^[25,28,30,32,33]^ Meta-analysis was performed using a fixed-effects model (*P* = .99, *I*^2^ = 0%). The results are shown in Figure 6, and the test group improved the FA of the CST across the infarct zone better than the control group [MD = 0.04, 95% CI (0.02, 0.06), *P* = .0002].

#### 3.5.5. ADC.

**Figure 7.**
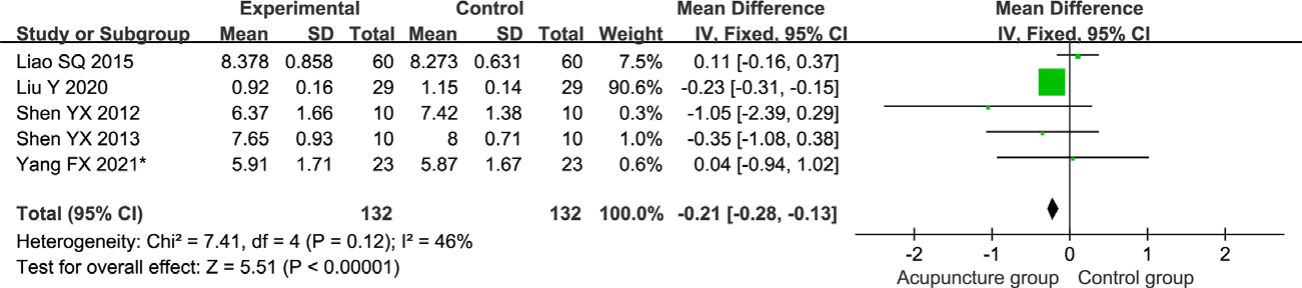
Meta-analysis of the average diffusion coefficient.

Five studies reported ADC values for the affected cerebral peduncle, including 132 patients in the test group and 132 patients in the control group.^[25,27,28,30,34]^ Meta-analysis was performed using a fixed-effects model (*P* = .12, *I*^2^ = 46%). The results are shown in Figure 7, the ADC values of the affected cerebral peduncle were higher in the test group than in the control group [MD = −0.21, 95% CI (−0.28, −0.13), *P* < .00001].

### 3.6. Assessment of publication bias

**Figure 8.**
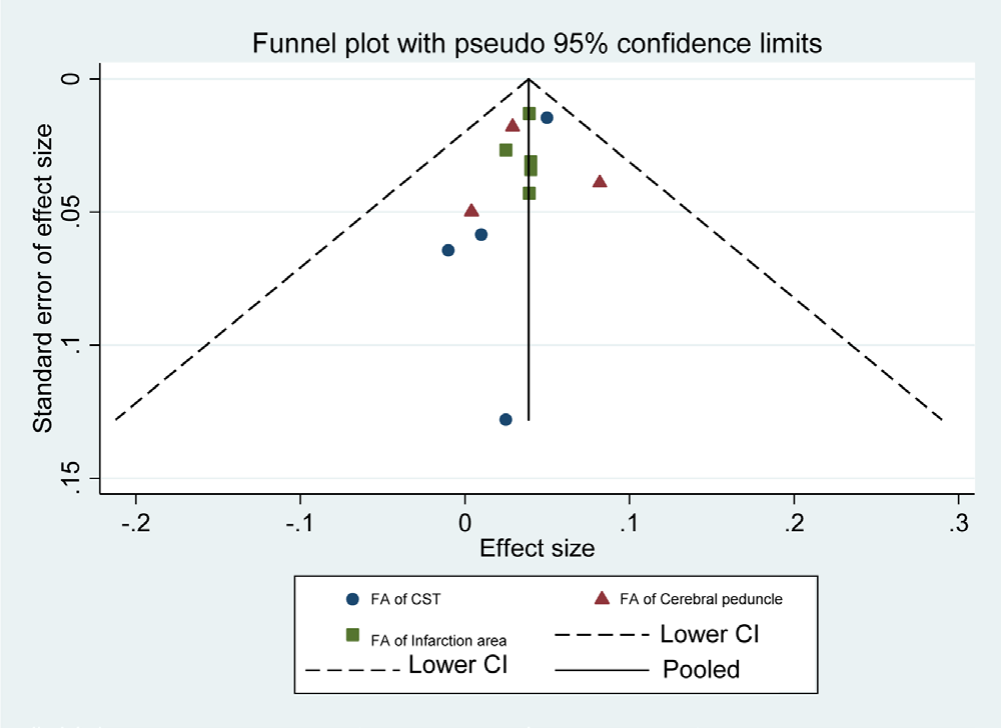
Funnel plot of the fractional anisotropy. CST = corticospinal tract, FA = fractional anisotropy.

Funnel plots can be used to assess the presence of publication bias in the observed data and to assess publication bias in FA. As shown in Figure 8, the left-right distribution of the scatter points is more asymmetric, indicating a slight publication bias in the included studies.

## 4. Discussion

Stroke is currently the leading cause of death from disease in China. Motor dysfunction after IS is mainly due to abnormal motor function caused by damage to upper motor neurons and the absence of central neural control of the motor system in the cerebral cortex. In China, acupuncture therapy combined with conventional therapy or rehabilitation is increasingly used in the long-term treatment of motor dysfunction recovery after IS. The rationale for acupuncture therapy is not well understood, and because of the obvious differences between traditional and modern medical theory, the role of acupuncture therapy needs further comprehensive and systematic evaluation. It is currently believed that acupuncture treatment improves circulation and metabolism, and protects the vascular endothelium for circulatory perfusion, thereby reducing tissue edema, decreasing the content of calcium ions in brain tissue, avoiding or reducing neuronal necrosis, promoting a significant improvement in the ultrastructure of neuronal mitochondria in the cerebral ischemic area, and causing a significant increase in the number of neurons in the brain, significantly inhibiting ischemic neuronal apoptosis, thereby promoting the functional recovery of brain tissue, and improving the coordination of the active, antagonistic, and synergistic muscles of the affected limbs through the bidirectional regulation of acupuncture, which in turn also improves the motor function of the patient.^[36]^ However, the current evaluation of the efficacy of acupuncture is mostly performed using subjective parameters, which lack objectivity, while inconsistent criteria for evaluation lead to inconsistent judgments of the efficacy of acupuncture.^[37]^ Multimodal MRI is currently a noninvasive means of objectively evaluating the role of needling in patients with cerebral infarction. Therefore, the present study included studies using DTI as a detection tool, which allows the use of objective factors to evaluate the effect of needling by excluding subjective factors. To our knowledge, this is the first systematic review and meta-analysis based on DTI to evaluate the efficacy of acupuncture on motor dysfunction and CST remodeling after IS. CST is the most important downstream motor conduction fiber connecting the cortical motor area and spinal cord neurons.^[38]^ Previous pathological studies have shown secondary injury and degeneration in distal nerve fiber regions downstream of the lesion, in addition to the focal area, after cerebral infarction.^[39]^ Among these, damage to the corticospinal tract is closely related to the recovery of distant motor function in patients.^[40]^ In the early stages of acute ischemic stroke, subtle changes in FA of the corticospinal tract downstream of the lesion can be detected using DTI imaging, which is useful for observing the extent and dynamic evolution of degeneration occurring in the corticospinal tract.

In the assessment of patients’ clinical performance, we selected NIHSS, BI, and FMA as indicators for evaluation. As an evaluation of the degree of neurological deficit, the NHISS has the best reliability and validity criteria.^[41]^ Although the NIHSS can quickly make a judgment about the degree of neurological deficit after stroke, the scale is less sensitive to reflect the patient’s coordination, gait impairment, cortical sensory function, and distal motor function.^[42]^ Therefore, we combined the BI and FMA to objectively represent the efficacy of acupuncture treatment in improving motor function in patients. The BI assessment is simple, with high reliability and sensitivity, and is currently the most widely used disability scale in the world to predict treatment outcomes, length of hospital stay, and prognosis.^[43]^ The scale is used to evaluate the degree of neurological disuse by the completion of daily living abilities in patients with cerebral infarction, and it can be used for prognostic speculation in the acute phase of stroke.^[44]^ In patients after IS, the most common motor dysfunction is in the upper and lower extremities,^[45]^ and the FMA can be used to infer motor performance and quality of movement in individuals with different degrees of chronic post-stroke injury and is particularly good at assessing motor performance in the upper and lower extremities.^[46]^ In this study, we found that acupuncture interventions improved patients’ NIHSS compared to conventional treatment or rehabilitation. Three of these studies showed that patients improved better than controls in NIHSS just after 2 weeks of acupuncture treatment. This suggests that acupuncture interventions may have a positive effect on neurological improvement in a short period. Three studies reported improvements in limb movements after acupuncture at 2 and 4 weeks, with one study finding better NIHSS, FMA, and BI than the control group after 3 months of follow-up. Four studies reported better improvements in BI scores in patients than in the control group after 2 weeks of acupuncture treatment. This suggests that the patient’s ability to perform daily living activities with acupuncture interventions may be better recovered with the aid of acupuncture in combination with rehabilitation. In conclusion, the improvement of clinical performance of acupuncture in patients with motor dysfunction in IS is positive.

For the improvement of brain microstructure, this study focuses on summarizing the studies related to observing the changes of corticospinal tracts under pinpoint intervention after cerebral infarction. Currently, DTI is the only noninvasive imaging method that can display the white matter fiber tracts of the brain in vivo. DTI can detect lesions of the CST in IS and can visually and clearly show the compression, tortuosity, and deformation of the CST, as well as clarify the relationship between the lesions and the white matter fiber tracts. The observation of nerve fiber bundle lesions and the appearance of functional impairment is important to clarify the exact anatomical location of the lesion and to assess the function.^[47,48]^ The anisotropy in the cerebral cortex is low, while the anisotropy in the white matter is high, and the more compact the white matter fiber bundles are, the more significant the white matter anisotropy is. The microstructural damage to brain tissue caused by IS lesions is mainly in the central and limbic brain tissue due to ischemia and hypoxia, with secondary inflammatory reactions causing degeneration of axons and myelin sheaths of nerve fiber bundles or secondary degeneration of axons and myelin sheaths of fiber bundles distal to the lesion, resulting in a decrease in white matter fiber bundle anisotropy.^[49]^ The various anisotropies of diffusion can be achieved by quantitative metrics, such as FA, ADC, and MD. Currently, the most commonly used parameter for quantification is FA.^[50]^FA images provide better gray-white matter contrast and easy identification of lesion sites, making the measured FA more accurate. Also, FA does not change with the direction of rotation of the coordinate system, and FA is the physical characteristics of the tissue, and the values obtained at different times, with different imaging devices, and between different subjects for the same object are comparable.^[51]^ Li et al found that there was no significant difference between the treated and control groups in the mean FA values of the whole-segment CST after acupuncture treatment, which may be since the mean values of the whole-segment fiber bundles do not fully reflect the specific details of the changing CST remodeling, which may obscure potentially important information.^[52]^ Therefore, the cerebral peduncle was additionally selected as the primary anatomical location for this study. The long axis of CST in this area is vertical, which can ensure the integrity of the target spinal tracts; the CST in this area are closely arranged and easily distinguishable from other neighboring fiber tracts; the fibers are reconstructed by applying 2 ROIs at the same time are the fibers that pass through both areas, which ensures the authenticity of the reconstructed fiber tracts. In this study, after 4 weeks of treatment, the FA values in the corresponding areas of the treatment group increased compared to the control group, suggesting that nerve fiber bundle reorganization and repair occurred, indicating that after 4 weeks of acupuncture combined with conventional rehabilitation treatment resulted in nerve fiber bundle remodeling, axon regeneration, and thus functional recovery in the lesion area. Yang et al used the DTI technique to visualize some areas through which the CST passes and evaluated the relevant parameters FA and ratio of FA and found that both the acupuncture and control groups improved the spatial structural remodeling of the CST in the infarct focus area and the affected posterior limb of the internal capsule, cerebral peduncle, and pons, and both correlated with motor function, but the improvement was more significant in the acupuncture group. This is consistent with the findings of many international rehabilitation scholars.^[28,52]^

ADC has good sensitivity and specificity for the diagnosis of cerebral infarction.^[53]^ Since changes in ADC are related to the movement of water molecules in the body, it can describe the diffusion of water molecules in the body, and different levels of values occur at different times of cerebral infarction. The progression of stroke can now be distinguished by monitoring changes in ADC.^[54]^ Usually, there is a significant decrease in ADC at the beginning of cerebral infarction, followed by a gradual increase as the disease progresses, and finally, it can reach near normal tissue levels.^[55]^ In this study, the comparison of ADC at the cerebral peduncle was found to be higher in the acupuncture group than in the control group, reflecting the possibility that acupuncture intervention may more significantly improve the dispersion of water molecules. All 5 studies included in this index had treatment periods of more than 2 weeks, with the longest reaching 1 month, and the ADC elevation may have also increased gradually with the duration of the disease. It was noted that ADC values on the focal side increased and decreased before and after acupuncture treatment, which may be due to the increase in neuronal myelin degeneration and axonal lysis, but the cellular debris generated by axonal disintegration can prevent the random diffusion of water molecules, which, together with glial cell proliferation, further restricts the diffusion of water molecules and results in a decrease. As the disease progresses, glial cell proliferation reaches a stable state and the disintegrating cellular debris is cleared by the body, the degree of water molecule diffusion restriction decreases, and ADC increases.^[25,56]^ Therefore, it is suggested that the treatment time of 2 weeks of acupuncture might be significant for the ADC at the cerebral peduncle of CST that is starting to improve. Shen et al^[34]^ concluded that ADC on the side of the lesion was lower than normal in the acute phase and gradually increased with the progression of the disease, equaling the normal value around the 4th week.

The systematic review found remodeling of corticospinal tracts under acupuncture intervention, a phenomenon considered to be related to the fact that acupuncture has mechanisms such as improving blood flow in ischemic areas of the brain, accelerating the establishment of collateral circulation,^[57,58]^ inhibiting the process of the inflammatory response,^[59]^ promoting neurogenesis and cell proliferation and anti-apoptosis in the central system, reducing myelin damage and promoting myelin regeneration, and increasing the plasticity of nerve fibers,^[60]^ thereby promoting the self-repair of damaged CST structures and promoting an increase in the number of fiber tracts. Lan et al^[61]^ showed that acupuncture in a rat IS model significantly increased the local anti-inflammatory effect of stroke, and they suggested that it could exert neuroprotective effects by inhibiting the release of local cellular inflammatory factors to achieve control of the inflammatory response process. Some studies have suggested that acupuncture can reduce oxidative stress induced by cerebral ischemia,^[62]^ such as Guo et al,^[63]^ who found that acupuncture has an antioxidant effect on cerebral ischemic injury by inhibiting NOX-mediated oxidative damage through acupuncture intervention in mice with cerebral ischemia, thus achieving neuroprotection. Acupuncture also increases brain-derived neurotrophic factor (BDNF) and vascular endothelial growth factor (VEGF). BDNF/VEGF are important nutritional mediators for the survival of neural stem cells and they stimulate the growth of new nerves and the migration of neurogenic regions. Tian et al^[64]^ after stimulating MCAO rats with electroacupuncture found that acupuncture facilitated stem cell differentiation after cerebral ischemia, increased BDNF and VEGF expression, and upregulated neuroprotective substances. Acupuncture also promoted cell proliferation in ischemically injured tissues. It promotes the proliferation of astrocytes and neural stem cells by activating the Wnt/β-catenin cell signaling pathway^[65]^; in addition, acupuncture also increases the expression of cell cycle proteins by upregulating stem cell factor, c-Kit gene, matrix metalloproteinase 9 and mRNA expression.^[66]^ In conclusion, acupuncture can exert neuroprotective effects and promote the remodeling of the corticospinal tract through multiple pathways after IS, and the underlying mechanisms still need to be explored in further basic experimental studies.

This systematic review accurately and objectively presents indicators related to corticospinal tract remodeling in patients with IS after acupuncture treatment with the aid of DTI technology, but there are still some limitations to consider; first, all 12 eligible studies declared randomization, but 5 studies did not specifically describe the randomization method. Of all eligible studies, only 4 described specific allocation concealment methods, and the setting of blinding in acupuncture studies is critical and difficult, which may also lead to selection bias in the results. Since the studies included in this systematic review were studies related to the combination of multimodal MRI and acupuncture, their study design and methodology were relatively new, thus resulting in a small number of included studies. There is no sample size calculation method for studies related to the elucidation of the intrinsic mechanisms of acupuncture treatment with the help of multimodal MRI, and the sample sizes of the included studies were generally insignificant. The application of DTI has not been popularized in clinical treatment, and it is only widely used in clinical research. This may be the reason why there are few relevant clinical scientific research. In addition, differences in the analysis process and measurement methods of DTI may have some influence on the results. Therefore, Studies with multicenter and large samples of RCTs are lacking in this systematic review, thus affecting the strength of evidence and level of recommendation. Second, the control group was treated conventionally without specifying differences in the type, dose, and frequency of specific drugs, leading to increased clinical heterogeneity. The inclusion criteria did not specify the patient’s infarct area, and the duration and severity of stroke may vary, which also increases clinical heterogeneity. Finally, the aim of treatment is still to improve the long-term prognosis of the patient’s motor function. 2 studies were performed with follow-up, and most of them have not yet clarified the long-term efficacy of acupuncture combined with conventional treatment. Currently, because clinical studies of acupuncture treatment involve the diversity of intervention modalities and the specificity of acupuncture points, improving the design and implementation of high-quality clinical studies of acupuncture is the key to improving the level of clinical evidence. In future studies, the design of such acupuncture clinical studies should be strictly standardized, such as reasonable sample size, more rigorous methodological design, precise randomization methods, implementation of allocation concealment and blinding, use of sham acupuncture controls, and encouraging the publication of negative results.

## 5. Conclusion

Existing evidence shows that acupuncture combined with conventional treatment can prove the beneficial effect of improving motor dysfunction in patients after IS, promoting the remodeling of the corticospinal tract, and significantly improving the NIHSS, FMA, BI, FA, and ADC. However, due to the overall quality of eligible studies, more rigorous design, and standardization, high-quality randomized controlled trials are expected to further validate the clinical role of acupuncture in promoting the corticospinal tract.

## Author contributions

**Data curation:** Hailun Jiang, Jieying Zhang.

**Project administration:** Shizhe Deng, Zhihong Meng.

**Visualization:** Boxuan Li, Qingqing Jia.

**Writing – original draft:** Weiming Zhu.

**Writing – review & editing:** Zhihong Meng.

## Supplementary Material




