# Supplementary material for: Assessment of corticospinal tract remodeling based on diffusion tensor imaging in the treatment of motor dysfunction after ischemic stroke by acupuncture: A meta-analysis
Source: Medicine (Baltimore). 2023 Aug 11;102(32):e34618. doi: 10.1097/MD.0000000000034618 (PMC10419801; doi:10.1097/MD.0000000000034618)
Supplement: Supplementary file 2 [file medi-102-e34618-s002.pdf]

Supplementary Table 2: Searching strategy and searching terms.

1	Acupuncture
2	Electroacupuncture
3	Pharmacopuncture
4	Needle
5	Stroke
6	Apoplexy
7	Cerebral vascular accident
8	Cerebral infarction
9	Ischemic Stroke
10	Diffusion tensor imaging
11	DTI
12	Acupuncture-related terms: 1 OR 2 OR 3 OR 4
13	Stroke-related terms: 5 OR 6 OR 7 OR 8 OR 9
14	Neuroimaging-related terms: 10 OR 11
15	Final searching terms: 1 AND 12 AND 13 AND 14
